# Local Community Perspectives on the Role of Institutional Stakeholders in Advancing Forest Landscape Restoration in Ghana

**DOI:** 10.1155/sci5/3179536

**Published:** 2026-05-30

**Authors:** Yaw Appau, Mercy Afua Adutwumwaa Derkyi, Anthony Baidoo

**Affiliations:** ^1^ Department of Forest Science, University of Energy and Natural Resources, P.O. 214, Sunyani, Bono Region, Ghana, uenr.edu.gh; ^2^ Department of Plant Development, Center for Plant Medicine Research, P.O. 73, Akuapem-Mampong, Eastern Region, Ghana; ^3^ School of Environmental and Natural Sciences, Bangor University, Gwynedd, UK, bangor.ac.uk

**Keywords:** collaboration, communities, governance, institutions, landscape, perception, restoration

## Abstract

The study explored community views on the roles of various institutional actors in promoting forest landscape restoration in Ghana. Using mixed methods, data were gathered from 417 households in the Kakum Conservation Area through surveys, interviews, and focus group discussions involving representatives from the Forestry Commission (FC), the Cocoa Board (COCOBOD), licensed buying companies (LBCs), nongovernmental organizations (NGOs), and traditional authorities. Results show that NGOs are viewed as the most effective due to their community engagement, responsiveness, and empowerment efforts. Meanwhile, the FC, COCOBOD, and LBCs received lower ratings due to limited interaction and perceived inefficiencies. Traditional authorities are vital in mobilizing communities, enforcing cultural conservation, and safeguarding forest resources, highlighting the significance of informal governance. Factors such as demographics, visibility, engagement frequency, past performance, and credibility shape community perceptions of these institutions. Additionally, 98% of respondents noted weak or nonexistent coordination among institutions, often due to conflicting goals and competition, especially between NGOs and LBCs. The study emphasizes that integrating traditional and formal governance systems is essential for sustainable restoration. Strengthening institutional collaboration can enhance restoration outcomes, but state institutions should remain central, with activities tailored to local needs.

## 1. Introduction

Achieving sustainable forest utilization has necessitated several governance transitions, especially in the tropics, due to outdated policy frameworks that fail to foster sustainable forest management [1, 2]. This paradigm shift has been facilitated by international forest frameworks, including the Rio Declaration, the Convention on Biological Diversity, the UN Declaration on the Rights of Indigenous Peoples, and the Reducing Emissions from Deforestation and Forest Degradation (REDD+), as well as Forest Law Enforcement, Governance, and Trade (FLEGT). A common feature of these policies is their pursuit of partnership, collaboration, and engagement in sustainable forest governance, thereby addressing the diverse needs and interests of stakeholders [3, 4].

Forest landscape restoration (FLR) has been a highly touted and widely applied forest conservation tool in global efforts to reverse forest degradation over the last decade [5]. This is consistent with the forestalling of ecosystem functions by enhancing biodiversity conservation, rural livelihoods, and carbon sinks [6], as promoted by the Bonn Challenge. FLR encompasses multiple dimensions of forest conservation, including economic, social, and ecological outcomes [7]. FLR drives landscape‐level restoration [8], not only to fulfill reforestation or afforestation objectives [9] but also to meet human welfare needs [10]. Governance mechanisms that foster the inclusiveness of diverse actors’ interests are crucial for achieving positive outcomes [11].

The delivery of successful restoration outcomes centers on institutions that play diverse roles in restoration efforts, including capacity building, funding, and monitoring [12, 13]. Nonetheless, the performance of these institutions is primarily evaluated through a policy or institutional lens, or by how well they have achieved their objectives [14], without reference to how local communities, a key factor in landscape restoration, perceive their role or performance. It is suggested that excluding the local people’s perspective on institutional performance does not enhance institutional efficiency and serves as a basis for nonparticipation [15]. Although Ghana has made significant strides toward these global commitments (the Bonn Challenge and the UN Decade of Ecosystem Restoration), little is known about how communities perceive the contributions, limitations, and coordination of institutional stakeholders. Meanwhile, fragmentation and weak interorganizational linkages are commonly identified as obstacles in FLR initiatives [16]. This limitation could exacerbate the mismatch between restoration efforts and local realities, leading to adverse outcomes in future restoration projects.

The study makes three main theoretical contributions. First, it expands participatory governance theory by highlighting how community perceptions affect evaluations of institutional performance and local involvement in restoration efforts. It also improves environmental perception theory (EPT) by demonstrating that perceptions are part of a feedback mechanism linking institutional actions, community trust, and restoration outcomes. Additionally, the research provides fresh insights into polycentric governance. These contributions aim to create a more complete framework for understanding governance dynamics in community‐driven restoration initiatives.

Consequently, the study aims to explore how local communities perceive the roles, responsibilities, and effectiveness of institutional stakeholders involved in FLR initiatives. The study, therefore, addresses the following questions: (1) What specific roles do local communities attribute to institutional stakeholders? (2) In what ways do communities assess the responsibilities of institutional stakeholders in FLR? and (3) What factors influence these perceptions?

## 2. Conceptual Framework

**FIGURE 1:**
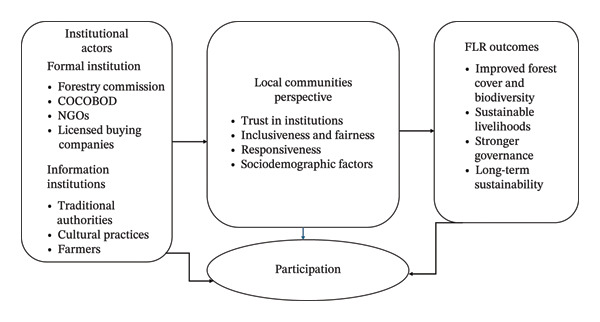
Conceptual framework for the study.

In this study, we are guided by the interrelations between institutional actors, household perceptions, and FLR outcomes (Figure 1). The analysis is based on the premise that institutions play a significant role in achieving sustainable restoration outcomes. At the same time, perceptions of institutional roles among local communities are among the major drivers of participation, compliance, and long‐term community engagement in FLR initiatives. Institutional actors shape community perception through their performance in FLR initiatives. These perceptions inform the level of community participation, which, in turn, influences restoration outcomes. A feedback loop connects restoration outcomes to institutional performance and community perceptions, underscoring the dynamic, iterative nature of FLR governance (Figure 1).

## 3. Materials and Methods

### 3.1. Research Design

A mixed‐methods case study design, employing an explanatory approach, was used to explore the role of institutional actors in shaping FLR from the perspective of local communities. The case study design with an explanatory approach is appropriate because FLR governance is embedded in complex institutional and social‐ecological systems that require a comprehensive understanding [17]. A mixed‐methods approach integrates quantitative data from communities on the perception of institutional performance in FLR with qualitative insights from institutional actors, thereby providing a nuanced analysis [18].

### 3.2. Study Area

**FIGURE 2:**
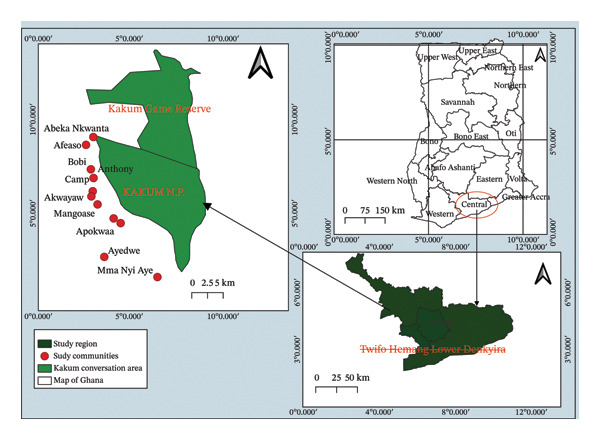
Map of Ghana showing the study area (authors’ construct).

The study was conducted in the Kakum Conservation Area (KCA), situated between Twifo‐Hemang Lower Denkyira and Assin South Districts in Ghana’s Central Region. The KCA includes Kakum National Park and the Assin Attandanso Reserve (AAR) (Figure 2). It was initially established as a forest reserve in 1931 and was later designated as Kakum National Park in 1991 under the Wildlife Reserves Regulations (LI 1525) (https://fcghana.org/kakum-national-park-assin/, accessed on October 30, 2024). Covering an area of 350 km^2^, the KCA features a characteristic moist evergreen rainforest, located between latitudes 5°20′ and 5°40′ and longitudes 1°18′ and 1°26′ W. The KCA is internationally recognized as one of the intact biodiversity areas within the Upper Guinean hotspot regions in West Africa [19]. It is among the most developed and reputable ecotourism destinations, known for its rich biodiversity (Ibid.). About 200 species have been documented within the reserve, including five hornbill species, Fraser’s eagle owl, and African gray and Senegal parrots. Currently, over 400 butterfly species have been recorded. The area is home to more than 40 species of wild animals, including bongos, elephants, and red river hogs, as well as a diverse bird population (Ibid.). The region is also known for rubber plantations, palm oil production, and cocoa farming. Additionally, the park significantly contributes to the local economy owing to its popularity as an ecotourism hub [20] (Figure 2).

### 3.3. Sampling Procedure

Sampling is essential for determining the population of interest in a study and its representation, thereby enabling statistical inference about the population [21]. The sampling was conducted in a stepwise approach to capture all relevant samples for the study. The first step was to select the KCA as the study area, which was informed by the uniqueness of the context it presents: a mix of agricultural and protected landscapes, followed by the identification of communities that have been engaged in restoration projects, ensuring that respondents had direct knowledge of the performance of institutions in restoration governance, resulting in 11 selected communities. We employed systematic random sampling to select 420 families that have participated in restoration activities supported by institutional stakeholders. The process was completed by selecting respondents, who included household heads or adult family members. Key institutional actors were selected through purposive sampling because of their specific roles in FLR. This included officials from the Forestry Commission (FC) and the Cocoa Board (COCOBOD), traditional leaders, and representatives of NGOs.

### 3.4. Data Collection

Data collection was preceded by a thorough explanation to participants of the study’s purpose and the rationale for seeking their input. Ethical procedures were strictly adhered to throughout the study. Verbal consent was obtained from all participants after clearly explaining the study’s aims, their right to withdraw at any point, and the voluntary nature of participation. To ensure anonymity, we did not collect respondents’ personal data during interviews, surveys, and focus group discussions. Transcripts were manually coded using anonymized labels, and responses were handled to prevent any link to individual participants. These measures ensured confidentiality and protected participants’ identities during data handling and analysis.

We complied with all community entry protocols and ensured the data collection process was free of actions that destabilized community cohesion.

A sequential data collection approach were adopted; quantitative data were collected first, followed by qualitative data through a household survey, and then by subsequent interviews and focus group discussions. A household survey was conducted in the selected communities to understand the local people’s perspective on the role of institutions in FLR. We engaged with 417 households, comprising both male‐ and female‐headed households, as well as other household members. The data were collected using a pretested structured questionnaire. The questionnaire consists of four sections: (1) household demographics; (2) roles of institutions in FLR governance; (3) perception of institutional stakeholders; and (4) institutional collaboration.

The study employed structured questionnaires that combined open‐ended and closed‐ended questions [22]. The closed‐ended questions, which were Likert‐style (1–*very poor*, 2–*poor*, 3–*fair*, 4–*good*, 5–*very good*), were useful for measuring people’s perceptions of institutional actors’ performance in FLR. The open‐ended questions allowed respondents to freely express their experiences and suggestions. The Likert‐style approach to data collection ensures standardization and easy analysis due to its structured format [23]. By using these questions, the study captures nuanced data on local communities’ agreement or disagreement with stakeholder performance in FLR. In addition, it consolidates data into measurable indicators that enable statistical comparisons across groups, a critical approach for evaluating the performance of multiple stakeholders involved in FLR initiatives. Grimmond et al. [24] noted that Likert‐style questions limit subjective interpretation and external influences on responses, thereby reducing potential response bias. This question type is essential, especially when assessing an institution’s performance, as it reduces social desirability bias, in which participants may alter their responses to suit their personal expectations [25]. The household surveys were administered through face‐to‐face interviews, assisted by trained field assistants who used the local language to eliminate language barriers.

Institutional representatives were interviewed using a semistructured interview guide. The interview guides were used to explore stakeholder roles, interactions with communities, challenges faced, and perceptions of governance outcomes. Interviews provided in‐depth insights into institutional perspectives, complementing community views obtained from the household survey.

### 3.5. Data Analysis

The data were cleaned for consistency and completeness by eliminating duplicate entries and confirming the data source [26]. We employed frequencies, means, and percentages to summarize household responses, including perceptions, demographics, and other relevant information, with SPSS Version 30. Multiple linear regression models were used to examine the relationship between respondents’ characteristics and their perceptions of the roles played by key institutions in FLR. The general model is specified as follows:

(1) Ratingi=β01+β.Educationi+β2.Durationi+β3.Agei+β4.Sexi+β5.Occupationi+β6.Residentiali+∈i,

where Rating_*i*_: perception score of respondents regarding the role of a specific institution (1–5 scale), *β*_0_: intercept, *β*_0_ − *β*_1_: coefficients for each independent variable, ∈_*i*_: error term

The Likert‐scale perception scores (1–5) were analyzed as continuous variables when calculating means and in the multiple linear regression models. This approach is common in perception research, but it has a drawback [27]: Likert responses are ordinal and do not inherently have equal intervals between categories. Consequently, treating these ratings with linear methods may not fully represent their ordinal nature. The qualitative data were manually coded using an iterative thematic analysis following Isangula et al. [28]. After transcription, the authors independently reviewed the interview and FGD transcripts multiple times to become familiar with the content. They then manually generated the codes based on recurring themes related to institutional roles, community perceptions, and governance outcomes. These codes were grouped into broader categories through constant comparison, helping identify new, converging, and opposing viewpoints. To ensure the themes were consistent, accurate, and reflective of respondents’ perspectives, the authors collaboratively refined them. This manual, researcher‐driven coding process facilitated the emergence of context‐specific insights and allowed for triangulation with the quantitative findings [28].

The findings from the household survey were triangulated with the qualitative data, providing deeper insight into policy direction to strengthen community‐institutional collaboration in FLR.

## 4. Results

### 4.1. Household Demographics

A total of 417 households took part in this study. Of these, 73% (*n* = 304) were male‐headed households, and 27% were female‐headed households. Respondents’ ages ranged from 29 to 70 years, with an average of 48 years. The majority (31%) have lived in the area for at least 20 years, while 27% (*n* = 111) have resided there for 21 to 30 years. Only 5% have lived in the area for less than five years (see Table 1). Natives comprise a significant portion (55%) of the households, with 45% being migrants seeking sustainable livelihoods. Approximately 44% of respondents have no formal education, 30% have completed primary education as their highest level, and 2% hold a tertiary qualification. Most households (54%) primarily produce crops, with 45% working as cocoa farmers (see Table 1).

**TABLE 1:** Demographic characteristics of households.

Respondents background	Number of respondents	Percentage (%)
Sex		
Male	304	73
Female	113	27
Age		
29–40 years	90	21
41–50 years	141	36
51–60 years	145	35
61–70 years	33	8
Duration of stay		
< 5 years	22	5
6–10 years	95	23
11–20 years	130	31
21–30 years	111	27
> 30 years	59	14
Residential status		
Native	231	55
Migrant	186	45
Level of education		
Primary	123	30
JHS	76	18
SHS	26	6
Tertiary	7	2
No formal education	185	44
Primary occupation		
Crop production	225	54
Cocoa production	191	45.8
Oil palm plantation	1	0.2

### 4.2. Roles and Ratings of Formal Institutions in FLR

The study revealed that institutions are involved in seedling supply, training, supervision, monitoring, and addressing problems related to restoration activities (Table 2). The findings indicate that the FC did not train communities in restoration‐related activities and that such roles accounted for 14% of all such roles. COCOBOD carried out all roles, with the highest contribution in seedling supply (11%), accounting for 24% of the total roles indicated, reflecting the integration of restoration practices within the cocoa sector.

**TABLE 2:** Roles played by formal institutions in forest landscape restoration.

Institution/roles	Responses		Ratings	
	Number of respondents^∗^	Percentage of respondents (%)	Mean	Std. deviation
Forestry commission			3.69	0.847
Seedling supply	186	8		
Supervision and monitoring of restoration activities	73	3		
Facilitate problem‐solving related to restoration	74	3		
COCOBOD			3.98	0.539
Training on restoration practices	156	6		
Seedling supply	254	11		
Supervision and monitoring activities	104	5		
Facilitate problem‐solving	44	2		
Cocoa buying companies			3.79	0.581
Training	110	5		
Seedling supply	231	10		
Supervision and monitoring	139	6		
Facilitate problem‐solving	39	2		
Nongovernmental organizations			4.68	0.549
Training	237	10		
Seedling supply	241	11		
Supervision and monitoring	205	9		
Facilitate problem‐solving	200	9		

^∗^Multiple response.

Meanwhile, 23% of the roles were played by the Cocoa Buying Companies, with a leading role in seedling supply (10%). The study shows that nongovernmental organizations (NGOs) played an instrumental role in landscape restoration (39%), with the highest contribution in seedling distribution (11%) (Table 2). The high mean score (*M* = 4.68) indicates strong stakeholder recognition of NGOs’ central role in the implementation of restoration. The relatively low standard deviation suggests high consensus among respondents regarding the effectiveness and visibility of NGOs in delivering restoration‐related services.

**FIGURE 3:**
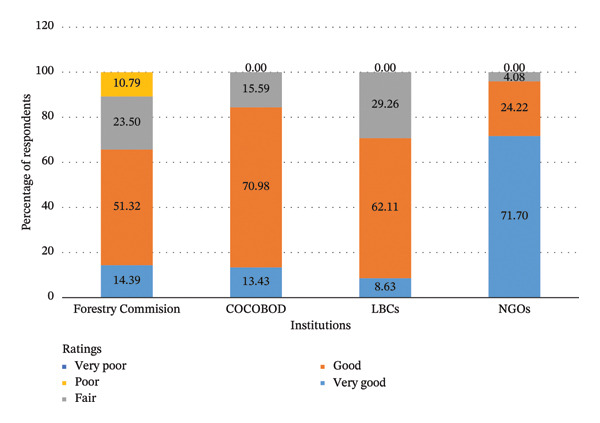
Rating of institutional roles by households.

Understanding how respondents view the institutional role of restoration practitioners is crucial for enhancing and coordinating effective implementation. The study shows that most respondents rated the role of NGOs as very good for restoration activities (mean = 4.68, SD = 0.549) (Figure 3). The roles of other institutions, including the FC, COCOBOD, and the cocoa buying companies, were rated as fair, with a mean range from 3.69 to 3.98 (Figure 3).

### 4.3. Respondents’ Perspectives on the Role of Chiefs and Traditional Leaders in FLR

The role of chiefs in restoration, as perceived by respondents, is presented in Table 3 below. The study revealed that the majority (29%; *n* = 258) of respondents noted that traditional authorities support restoration by enforcing cultural practices that conserve the forest. In comparison, 28% (*n* = 252) indicated that chiefs support mobilizing communities to engage in restoration activities. Twenty‐two per cent (*n* = 196) of the respondents mentioned that chiefs support restoration by preventing unauthorized land use in forest reserves. Lastly, their contributions were also felt in safeguarding against the illegal utilization of forest resources (21%, *n* = 191).

**TABLE 3:** Role of traditional authorities in forest restoration.

Roles of chiefs	Number of respondents^∗^ (*n*)	Percentage of respondents (%)
Enforcement of cultural practices	258	29
Support community mobilization	252	28
Prevent the misuse of forest resources	191	21
Prevent unauthorized land use in forest reserves	196	22

^∗^Multiple response.

### 4.4. Respondents’ View on Traditional or Cultural Practices Supporting Forest Conservation

The study revealed that cultural practices play a pivotal role in conserving forest resources within the study area. Most respondents (58%) cited “Taboo Day” as a significant practice that aids forest restoration, followed by myth (46%) and sacred grove (1%) (Table 4).

**TABLE 4:** Cultural practices supporting forest conservation.

Cultural practices	Number of respondents^∗^	Percentage of respondents (%)
Taboo day	310	53
Myth	270	46
Sacred grove	7	1

^∗^Multiple response.

Most respondents (*n* = 322, 77%) believed that cultural practices remain valid for forest conservation. In contrast, the remaining respondents (*n* = 95, 23%) indicated that cultural practices are not being adhered to, attributing the noncompliance to religion (*n* = 407, 75%), education, or de‐urbanization (*n* = 135, 25%).

### 4.5. Factors Influencing Institutional Ratings by Respondents

Table 5 presents the results of the factors influencing institutional ratings, as reported by respondents. The study found that respondents’ age was negatively associated with ratings of COCOBOD (*β* = −0.013, *p* < 0.001), NGOs (*β* = −0.013, *p* < 0.001), and traditional authorities (*β* = +0.048, *p* < 0.002). While younger respondents rated COCOBOD and NGOs highly, older respondents rated traditional authorities slightly lower. Male respondents rated the role of chiefs significantly higher than their female counterparts, although they rated COCOBOD marginally lower. The duration of stay was negatively predicted by ratings for the FC (*β* = −0.156, *p* < 0.001) and licensed buying companies (LBCs) (*β* = −0.070, *p* < 0.024). In contrast, it positively predicted the roles of traditional authorities (*β* = 0.0233, *p* = 0.002). Respondents’ educational level was positively associated with the FC (*β* = 0.101, *p* < 0.001), COCOBOD (*β* = 0.038, *p* < 0.013), and LBCs (*β* = 0.043, *p* < 0.009). However, these differences were not significant in ratings of NGOs and traditional authorities. Occupation is significantly associated with ratings of LBCs (*β* = 0.114, *p* < 0.036), NGOs (*β* = −0.112, *p* < 0.029), and traditional authorities (*β* = −0.0610, *p* < 0.001) (Table 5).

**TABLE 5:** Factors influencing institutional ratings by respondents.

**Variables**	**Forestry Commission**		**COCOBOD**		**LBCs**		**NGOs**		**Traditional authorities**	
	**β**	**p**	**β**	**p**	**β**		**β**	**p**	**β**	**p**

Intercept	3.774	0.001	4.464	0.001	3.793	0.001	5.257	0.001	0.593	0.001
Sex	0.002	0.981	−0.112	+0.060	+0.084	0.192	−0.031	0.606	+0.048	**0.002**
Age	0.001	0.914	−0.013	**0.001**	+0.003	0.486	−0.013	**0.001**	−0.0026	**0.010**
Duration of stay	−0.156	**0.001**	0.045	0.116	−0.070	**0.024**	+0.057	0.051	+0.0233	**0.002**
Educational level	+0.101	**0.001**	+0.038	**0.013**	+0.043	**0.009**	+0.008	0.611	−0.0018	0.654
Residential status	−0.001	0.993	0.019	0.744	+0.009	0.886	+0.013	0.824	+0.020	0.192
Main occupation	0.049	0.529	0.001	0.988	−0.114	**0.036**	−0.112	**0.029**	−0.0610	**0.001**
*R* ^2^	0.075		0.042		0.040		0.040		0.116	

*Note:* *β*: coefficients show the direction and magnitude of the effect. *p* *:* statistical significance at < 0.05. Bold values indicate statistically significant relationships. *R* ^2^: proportion of variance in ratings explained by the demographic predictors. COCOBOD: Ghana Cocoa Board. LBCs: Licensed Cocoa Buying Companies.

Abbreviation: NGO, nongovernmental organizations.

### 4.6. Perception of Respondents on Coordination Among Institutions in FLR

The study solicited respondents’ views on how different projects work together toward common restoration goals. Almost all the respondents (98%) indicated that coordination among different restoration projects is very poor and virtually nonexistent. The respondents’ view is that the institutions believe they are independent and do not require assistance from others to implement their projects. They also think that there is a lack of collaboration among the institutions because their objectives differ. They cited the different roles of LBCs and NGOs to support their assertion, emphasizing that LBCs are primarily involved in purchasing cocoa beans. In contrast, NGOs focus on capacity building and on providing access to their produce. Furthermore, they noted that some of the institutions view others as competitors. Moreover, they were optimistic that these institutions could find a common ground to work together for the good of the people.

## 5. Discussion

### 5.1. Institutional Roles in FLR

The findings reveal that various institutions assume distinct and sometimes overlapping roles in relation to FLR in Ghana, a characteristic example of polycentric governance, characterized by diverse levels of decision‐making and a degree of independence among the actors involved [29]. For instance, the FC serves as a regulatory authority, ensuring compliance with laws and policies governing forest resources through supervision and monitoring. The NGOs support communities in enhancing their understanding and facilitating restoration projects. COCOBOD, as a regulator in the cocoa value chain, determines yearly producer prices, while the LBCs act as middlemen between cocoa farmers and COCOBOD in the cocoa economy. They integrate restoration into the value chain to improve resilience and as a means of retaining their market share, respectively. The involvement of multiple institutions in restoration efforts reinforces the notion that single institutions cannot address the challenges posed by social‐ecological systems, such as those in landscape restoration [30].

The prominent involvement of NGOs across all roles highlights their strong commitment to community development [31]. Their participation in the landscape restoration supports the regulatory and oversight functions of the FC and COCOBOD, respectively. However, the limited engagement of state‐mandated institutions in addressing and resolving restoration‐related issues poses a significant governance challenge. This raises concerns about the long‐term viability of FLR efforts if facilitation remains primarily external, especially since NGOs’ presence is often temporal. While community members rate NGOs highly, this does not mean they should replace formal state and private agencies in leading FLR initiatives. NGOs generally operate under project‐based, time‐limited funding, which can affect sustainability. In contrast, the FC, COCOBOD, and LBCs are established legal entities state‐mandated with permanent structures crucial for regulation, monitoring, and ensuring continuity in restoration governance. Relying solely on NGOs risks undermines national accountability systems and weakens the institutional capacity at a landscape scale. Effective FLR demands a polycentric governance approach where NGOs complement, rather than replace, state and private institutions [32]. Both types of organization have unique, vital roles: NGOs foster community engagement and build capacity, while the FC, COCOBOD, and LBCs provide regulation, sector expertise, and supply chain support. Strengthening coordination among all actors is thus more effective than transferring leadership solely to NGOs.

Furthermore, the findings highlight the crucial role of NGOs in facilitating the shift to participatory governance at the local level and in providing communities with seedlings. This is reflected in their efforts in capacity building and facilitation, which support empowerment processes aimed at improving skills and confidence and enabling people to assert themselves in collective action [33]. The respondents view the FC as adopting a top‐down approach to forest restoration, supplying seedlings without engaging with them. This could risk sustainable community engagement in the absence of NGO support. Again, the positive perception of communities toward NGOs could be linked to the responsiveness of their interventions to local needs, thereby giving them legitimacy that can be leveraged for sustainable restoration activities. Governance theories emphasize that effectiveness improves when institutions are perceived as legitimate by local actors, foster greater cooperation, and reduce conflict [34, 35].

A feedback loop in the conceptual framework (Figure 1) shows how local people’s perceptions influence participation, which determines restoration outcomes and institutional ratings. In theoretical terms, this builds on participatory governance by illustrating that perceptions are not static but are iteratively shaped by factors such as past performance and credibility. Drawing on this, the study contributes to resilience theory in social‐ecological systems [36], which emphasizes the importance of incorporating community feedback into FLR initiatives to improve institutional roles and, eventually, meet people’s needs. This suggests that in a hybrid governance framework, actors do not simply co‐exist; they continuously interact, influenced by socio‐economic factors and institutional legitimacy. Additionally, these findings improve the understanding of feedback loops by showing that community perceptions are both an outcome of institutional actions and a driver for future institutional behavior. In this context, the EPT is expanded to illustrate that perception influences governance structures, impacting participation and restoration outcomes. Because of the ongoing relationship among past performance, perception, and participation, empirical evidence is likely to support the development of adaptive, hybrid governance models in FLR contexts. This enriches theoretical discourse by providing context‐specific evidence from Ghana, underscoring the need for participatory and polycentric approaches in FLR governance.

### 5.2. The Role of the Traditional System in FLR

The finding indicates that chiefs are instrumental in enforcing cultural practices, such as taboos, rituals, totems, and sacred groves, that prohibit harmful activities, including felling sacred trees or hunting in protected forests. This cultural enforcement creates intrinsic motivation for conservation and deters exploitation through spiritual sanctions, such as fears of ancestral retribution or illness [37], which are more effective in rural areas where state oversight is limited. For example, chiefs, as custodians of culture, ensure compliance with taboos on sacred trees, contributing to the preservation of biodiversity hotspots, such as sacred sites that host threatened plant and animal species [38]. These practices align with traditional ecological knowledge, which can reduce deforestation by restricting access and thereby encouraging regeneration [39]. In the Gurune‐speaking communities of the Upper East Region, chief priests (*tindaana*) enforce prohibitions against bush burning and indiscriminate logging [40], which are linked to spiritual harmony and thereby prevent habitat loss and soil erosion. This role supports broader conservation efforts by complementing statutory frameworks. For example, under the Wildlife Resources Management Act 2023, cultural practices enforced by chiefs help maintain the integrity of protected areas, as seen at the Boabeng‐Fiema Monkey Sanctuary, where taboos protect monkey populations and surrounding forests [41]. This typifies the institutional theory, which posits that integrating formal and informal structures yields positive outcomes [42].

Additionally, the findings suggest that chiefs play a crucial role in mobilizing the community for restoration. Their influence stems from their status as community leaders and the respect accorded to the chieftaincy institution in Ghana. Therefore, their involvement in a program increases social acceptance and encourages participation among their subjects. A typical example is found in the Gurune communities, where chiefs collaborate with NGOs and the FC in Community Resource Management Areas (CREMAs), organizing hunting associations and farmers for afforestation, thereby enhancing social cohesion and reducing resource conflicts [40].

Furthermore, respondents indicated that chiefs safeguard against resource destruction by leveraging their authority [38]. They mentioned that Chiefs, as custodians of lands under customary tenure in traditional settings, regulate land access, mediate land allocation, and enforce boundaries, preventing encroachment on forest lands for farming, which is crucial to reducing deforestation. In some instances, it is taboo to sell ancestral land, known as “dabo zuo,” in the Gurune culture [40], thereby preventing unauthorized sales, preserving forest integrity, addressing tenure insecurity that fuels degradation, and complementing government conservation efforts by reinforcing policies such as the Land Act 2020. For example, lower encroachment rates have been reported in areas where chiefs are active in resource management. At Kogyae Strict Nature Reserve, the chief’s intervention prevents land grabs, ensuring the harmonious and equitable allocation of land for sustainable agricultural practices and the maintenance of ecosystem services [43]. Similarly, in Indonesia, the use of social sanctions serves as a deterrent to forest encroachment [38]. The finding highlights the crucial role of chiefs in conserving Ghana’s forest resources, reinforcing the need for participatory forest governance. Their roles, including enforcing cultural practices, mobilizing people, safeguarding against resource misuse, and supporting formal efforts to curb forest depletion, align with SDGs 13 and 15.

The study advances institutional theory by proposing a hybrid governance model that integrates traditional authorities with formal state institutions to address gaps in participatory governance. Additionally, the study has demonstrated that informal systems provide intrinsic drivers that complement enforcement in areas with a weak state presence, reinforcing the significance of participatory governance. Theoretically, this finding suggests that traditional systems are as important as formal institutions in local‐level FLR governance, offering a sustainable path to conflict resolution and broad participation. This perspective shapes polycentric governance models, highlighting how overlapping roles among institutions can be hybridized to achieve positive outcomes rather than being fragmented.

### 5.3. Factors Influencing Households’ Perception of Institutional Roles

Understanding how personal characteristics (such as sex, age, educational level, residential status, duration of stay, and occupation) shape perception, particularly in conservation efforts, is essential for sustained participation.

Respondents’ sex was significantly associated with ratings of traditional authorities, which were positive and weak, indicating that males rated chieftaincy higher than females. The implication is that the male respondents align more closely with the roles of chiefs, likely because males dominate the traditional governance system. Historically, women are stereotyped as incompetent to assume leadership positions, particularly in a typical conservative setting. For instance, it is taboo for women in the Igbo tribe of Nigeria to be crowned as chiefs [44]. The weak rating could indicate that most respondents do not occupy traditional positions.

Age had a statistically significant negative impact on ratings of COCOBOD and NGOs. Younger respondents generally gave more favorable ratings to these institutions. This phenomenon may reflect generational differences in trust and acceptance of contemporary institutional mechanisms, or variations in the perceived relevance of institutional services, linked to individuals’ observations of related institutions’ activities over time. Furthermore, it is plausible that younger individuals are more frequently exposed to environmental education and communications that portray institutional roles in a positive light. Age was negatively associated with ratings; older respondents were somewhat more critical of chiefs, which could reflect greater exposure to traditional systems over time, leading to more nuanced or indifferent assessments.

In contrast, younger individuals may admire chiefs intensely, associating them with cultural identity and authority. The lack of relevance regarding the FC and LBCs may suggest that their views are less influenced by age differences, potentially underscored by the long‐standing, entrenched relationship between communities and the FC. There is limited public engagement with the communities, especially since respondents did not cite them for providing training or building their capacity on restoration activities. As noted in institutional theory, if mandated formal institutions, particularly state actors, are perceived as ineffective, individuals are less likely to recognize them and therefore regard them as rightful managers [45, 46].

The duration of an individual’s stay (length of time) in a community influences their ratings of the FC and LBCs, resulting in a negative association. However, there were significant positive ratings for the traditional authorities. The FC and the LBCs are notable institutions leading restoration programs. Given the regulatory nature of the FC’s role, a conflict has always existed between forest communities and the FC, which may help explain their slightly lower ratings. These residents might have prior experience with unsuccessful interventions or a deeper understanding of governance inconsistencies, which can lead to apathy toward these services. Similarly, the LBCs, as the primary contact for cocoa transactions, have direct and regular interactions with farmers. A highly polarized sector, plagued by misunderstandings and perceived misconduct by the LBCs that have persisted over time, could have influenced their negative ratings rather than their roles in landscape restoration. The findings align with the existing literature, suggesting that institutional trust may decline over prolonged exposure without observable, sustained effects [47, 48]. In contrast, the length of stay exerted a marginally positive influence on NGO evaluations. According to the EPT, people’s perceptions of environmental actors are shaped by their duration of engagement, experiences, and attachment to the environment [49].

Hence, longer‐term residents are more likely to rate positively because of their knowledge of past and current NGO projects, especially when these projects advance the community’s ecological and social goals. Nonetheless, a marginal rating is understandable, particularly when restoration results take time to materialize. This highlights NGOs’ crucial role in the forestry sector, particularly in restoring degraded landscapes. For example, Moluh Njoya et al. [50] noted that NGOs are essential in raising environmental awareness, promoting afforestation and reforestation, supporting sustainable forest management, and mobilizing communities for restoration efforts in Central Togo. Similarly, NGOs have maintained their presence in other parts of the world through consistent grassroots engagement, complemented by visible project outcomes. This finding reinforces the widely accepted importance of NGOs in forest conservation, including advocating for equity, building local capacity, and strengthening local governance mechanisms [51, 52]. The favorable rating of traditional authorities, attributed to the length of time respondents have lived in the community, suggests that these individuals have experienced and appreciated the value of traditional leadership in supporting forest restoration activities. Historically, as custodians of land, chiefs have played a key role in forest conservation by upholding cultural and spiritual practices that prevent deforestation. The involvement of chiefs is essential for effectively and efficiently managing Ghana’s lands and natural resources [53].

Educational level positively influences respondents’ ratings of COCOBOD and LBCs. In the context of EPT, an educated individual is more likely to appreciate their environment and the institutions that sustain it. A better understanding of their surroundings enables them to make informed decisions, especially when institutions such as COCOBOD and LBCs serve their interests [54]. Likewise, participatory governance theories highlight the role of education in shaping perceptions of institutions. It argues that individuals with higher education can engage in meaningful discussions, ask pertinent questions, and participate actively in decision‐making. Consequently, such individuals tend to be better informed, which is reflected in their ratings of institutional performance.

The primary occupation of respondents showed a significant negative correlation with evaluations of LBCs, NGOs, and traditional authorities regarding their roles in forest restoration. The indication is that certain occupations that depend directly on land, such as crop or cocoa farmers, receive slightly lower ratings than other professions. Accordingly, people’s perceptions of environmental outcomes and institutional mandates are shaped by their dependence on the environment and their experiences, as the EPT suggests. For instance, individuals whose occupations are directly impacted by restoration activities are more likely to engage with institutional policies and actions; if these actions fail to meet expectations, it often results in negative perceptions. Similarly, institutional theory posits that institutional integrity is maintained when its activities and visions yield mutually beneficial outcomes [55]. If the activities of institutions such as the traditional authorities, COCOBOD, and LBCs are perceived as not transparent and biased, other occupational groups may feel excluded, resulting in negative ratings. Institutional performance, therefore, involves not only achieving positive outcomes but also ensuring that the interests of all occupations are considered [56].

It is essential to emphasize that, in addition to household demographic influences on the perceived roles of institutional actors, other equally crucial factors were noted during focus group discussions, including visibility, frequency of engagement, previous performance, and the organization’s credibility, particularly in keeping its promises. The Wildlife Division of the FC is a state‐mandated institution responsible for managing forest resources. It is well known in the communities; however, its engagement with them is nonexistent. As captured: “*The only time you see them in the community is when they are passing information to us about the visit of their Bosses from Accra*.” Most respondents expressed dissatisfaction with their enforcement approach, which they described as selective. They alleged that the FC officials connived with strangers to illegally fell trees, but they were arrested, and the trees they had harvested for personal or community use were seized. However, the FC refuted these allegations: “*We are only doing our work as mandated by the act that established the Commission. Most of them are ignorant of the laws, hence, the wild allegations.”* In their engagement with the community, they acknowledge their shortcomings and attribute them to a lack of funds to embark on extension services. These findings raise fundamental issues relating to collaboration, enforcement, and capacity building, which are core components of the 2012 Forest and Wildlife policy [57] and the recent Wildlife Resources Management Act, 2023 (https://forestpolicy.org/sites/default/files/pdf/ghana.pdf; accessed on March 30, 2026).

The COCOBOD and the LBCs are mostly perceived negatively by the respondents, mainly because their roles involve financial transactions. Respondents perceive that COCOBOD is failing in its core mandate to improve cocoa farmers’ welfare, primarily due to low producer prices. As a respondent retorted, *“They are engaging in things that are not their duty, such as supplying us with tree seedlings; they should rather, together with the government, focus on fixing the right prices for our hard work.”* Hence, they are reluctant to engage in a restoration effort led by the COCOBOD. According to COCOBOD officials, they are doing their best to promote education on good agricultural practices (pruning, fertilizer and pesticide application, and pollination), and to support farmers with hybrid cocoa seedlings and tree seedlings to enhance their resilience to climate change.

Traditional authorities were highly rated for visibility and engagement. This is understandable because, as community leaders, they are often sought out for their presence and support before any restoration activity can occur. However, respondents were conflicted about whether they still have the legal authority to enforce restoration laws, especially since state laws take precedence over natural resource management under our current dispensation.

Building on EPT, which posits that perceptions of environmental actors are shaped by personal experiences and attachments. The study introduces demographic variables as key moderators influencing institutional ratings. Thus, the study extends the EPT by underscoring the importance of demographic variables, particularly age and education, in shaping ratings of restoration actors. This implies that demographic considerations should be embedded in FLR engagement to mitigate apathy. This insight offers a more robust theoretical lens for understanding the role of perception in driving participation in social‐ecological systems.

### 5.4. Institutional Collaboration

The study indicates that there is no collaboration among the various institutions in the restoration. Communities saw weak collaboration mainly caused by differing institutional mandates, power dynamics, and incentive schemes rather than communication issues. For example, state institutions, NGOs, and LBCs operate within a separate governance framework driven by regulatory authority, donor projects, and market competition, respectively. These varied goals make alignment challenging and obstruct coordinated restoration efforts [58, 59]. Communities also viewed competition for influence, visibility, and beneficiaries, particularly between NGOs and LBCs, as a significant barrier to working together.

One of the main reasons for the failure of institutional coordination is the dominance of powerful actors in decision‐making [60]. They tend to drive outcomes that serve their interests at the expense of the group’s goals. This is usually the case for state‐mandated institutions that shape policies. The FC’s dominant regulatory authority, combined with limited community engagement capacity, leads to a top‐down governance model that hampers horizontal collaboration [61, 62]*.* This shows how power relations influence whose opinions are valued, which goals are prioritized, and how resources are allocated. From a polycentric governance perspective, although multiple actors are involved, there are not enough interactions to foster collective efforts. This points to a structural governance gap that requires institutional reforms to align mandates, incentives, and accountability among actors. Such a system does not support integrated landscape restoration and conflicts with one of FLR’s core principles, which stresses stakeholder participation and participatory governance [63]. A hybrid governance approach, where institutions effectively coordinated their roles, provides a more sustainable path than the current disconnected system [64]. It is therefore crucial to coordinate institutions to align mandates, minimize effort duplication, and enable coordinated landscape‐wide planning. This approach improves accountability via state structures, enhances legitimacy through community engagement, and taps into private‐sector resources and efficiency. Additionally, fostering better cross‐sector collaboration with clear roles, shared decision‐making, and an integrated implementation framework will be vital for achieving resilient, inclusive FLR outcomes.

Mansourian et al. [65] noted that to ensure broad coordination, conflicts arising from differing interests, values, and power among institutional stakeholders require governance systems to address these power imbalances. Respondents noted that some institutions view one another as competitors, particularly the various LBCs and NGOs. Most LBCs used restoration projects as an incentive strategy to increase their market share in the cocoa trade; thus, any efforts by other institutions to implement similar projects are seen as a threat that could lure clients away. This competitive dynamic reflects deeper governance issues, including incentive misalignment and the absence of an overarching coordination framework to harmonize institutional roles across the landscape. This institutional mindset will hinder inter‐institutional collaboration, leading to duplication of efforts and potentially fostering long‐term apathy toward restoration activities.

Private‐sector institutions can improve collaboration by concentrating on common goals like climate resilience, cocoa sustainability, and community members’ livelihood enhancement. For instance, establish joint monitoring systems to avoid duplication and minimize unhealthy competition. Social innovations provide opportunities for private organizations to collaborate on sustainable restoration projects, such as shared incentive programs or co‐funding schemes. These social experiments can build trust, encourage organizational learning, and generate evidence to support more coordinated and scalable restoration efforts [66].

These findings offer a new theoretical perspective on polycentric governance, suggesting that broad consultation fosters resilience. In contrast, unchecked autonomy deepens the misalignment between institutional mandates and local needs, leading to the nonparticipation of restoration actors. By highlighting that collaboration requires a framework to align incentives, the study extends theories of multistakeholder partnerships and draws on the institutional interaction literature [67]. The study cautions against overreliance on external actors, as it raises sustainability concerns after the project’s expiration. Therefore, the study advances inter‐institutional collaboration, but with a strong state presence.

Another reason for the lack of cooperation was that the LBCs believed most NGOs were indirectly doing the bidding of other LBCs. There are instances where farmers have been instructed not to participate in NGO‐organized meetings, as they may be susceptible to influence and have been a significant source of tension between NGOs and LBCs at the local level. This is usually the outcome when institutions are not equally resourced. For instance, some institutions may have more financial and human resources than others, especially local institutions that may lack the capacity to contribute meaningfully and may be neglected, deepening existing power imbalances that hinder effective collaboration [68]. Differences in objectives and institutional autonomy complicate collaboration due to funders’ objectives, incentives, and differing implementation periods, underscoring the theoretical expectations in multistakeholder landscapes [69]. Indeed, FLR involves multiple actors with varying governance structures. It is worth noting that FLR institutions operate within a specific mandate and have limited incentives to work across sectors or scales [70]. For instance, policy misalignment arising from policy frames, objectives, instruments, implementation processes, and actors notably restricts institutional collaboration [71–73]. Accordingly, in Ethiopia, the FLR governance initiative was constrained by the dominance of the food security policy agenda, leaving little space for cross‐sector collaboration and consideration of other benefits, thereby keeping power within individual sector agencies [70].

Representatives of institutional actors agreed on the lack of collaboration but did not consider themselves competitors, as indicated by the communities. They mentioned that, although they are not collaborators, they do not undermine each other because each project is unique. They contribute their share to restoring the landscape and welcome more projects because they cannot do it alone.

In practice, institutional collaboration in the FLR initiative fails when a few institutions dominate decision‐making, mandates are fragmented, and there is no governance system to foster cross‐scale or sectoral collaboration [70]. Addressing these failures requires governance structures that integrate and manage power relations, strengthen inter‐institutional collaboration, and promote broad participation of all actors [60].

## 6. Conclusions and Recommendations

The study was conducted to understand how FLR is governed at the community level. It focused on the roles local people play and how they perceive them. The study reveals profound findings that shape our understanding of the interplay between communities and institutions in FLR governance.

People’s perceptions of the roles of institutional actors, including the FC, COCOBOD, NGOs, LBCs, and traditional authorities, are influenced not only by demographic factors but also by visibility, frequency of engagement, past performance, and credibility. The negative perception expressed by communities, especially for the COCOBOD and the LBCs, is often a result of perceived inequalities, deviations from core duties, and efforts that do not benefit farmers. This has led to mistrust over the years, and as a result, communities are distancing themselves from restoration efforts led by these institutions. The study revealed that traditional authorities are crucial actors in the restoration efforts. They are not just ceremonial heads; they also enforce traditional practices, such as taboos and the protection of sacred groves, which are biodiversity hotspots. They leverage their authority to mobilize the community for restoration activities and to institutionalize bylaws to safeguard against forest encroachment. Incorporating this informal governance system at the community level encourages comprehensive participation.

The weak institutional collaboration found in the study reflects deeper governance constraints, such as fragmented mandates, incentives, and power relations, and not just coordination or communication failures. Addressing these challenges requires governance systems that integrate all the institutional incentives and ensure cross‐sector collaboration at the community level.

Theoretically, the study advances our understanding of participatory governance by empirically demonstrating that participation in FLR is largely influenced by community perceptions of past performance, institutional visibility, and credibility. The study moves participatory governance beyond formal inclusion toward legitimacy‐based participation, as the findings show that perceptions influence trust and willingness to engage in FLR. Moreover, EPT is demonstrated by showing the central role of perception in shaping institutional action, community participation, and long‐term restoration outcomes. Rather than being static attitudes, perceptions actively reshape governance arrangements over time, influencing how institutions adapt their strategies and how communities respond. Furthermore, the study contributes new insights into polycentric governance by showing that hybrid governance is not a fixed institutional structure but an emergent process facilitated by interactions among actors in FLR. Given the important role of traditional authorities in FLR, it reinforces the integration of informal institutions into formal polycentric governance systems. Generally, these contributions offer a more dynamic and empirically grounded understanding of governance in FLR.

It is our recommendation that:

• The FC, as a matter of urgency, embarks on rigorous community engagement to improve the seemingly negative perception held by community members. As a state institution, the FC can advocate for hybrid governance by formally integrating traditional cultural systems into forest resource governance in Ghana.
• The COCOBOD should sustain the seedling distribution and training to improve farmers’ resilience in changing climatic conditions while leveraging the uniqueness of the landscape (cocoa‐forest landscape) for carbon markets for additional income to mitigate the mistrust linked to lower producer prices.
• LBCs should coordinate with COCOBOD and NGOs on restoration efforts, minimize duplicated competition, and implement transparent engagement practices to build stronger farmer relationships over time.
• The role of traditional authorities should be formalized within FLR by incorporating cultural conservation efforts and indigenous ecological insights. Utilizing their credibility and influence in the community can greatly boost participation, ensure compliance, and support the sustainable protection of restored landscapes.
• All relevant institutions should come together to create a multistakeholder coordination platform, develop common monitoring frameworks, and collaboratively design restoration incentives. This approach aims to reduce fragmentation and promote scalable, community‐aligned FLR outcomes.

### 6.1. Limitations of the Study

The study took place in the KCA, an area marked by strong traditional authority systems and long histories with restoration actors. Although this offers valuable insights into hybrid governance, the results may not be entirely applicable to other forest landscapes in Ghana or different regions with varying institutional, cultural, or ecological contexts.

Although the study points out weak institutional collaboration and explores its governance factors, it does not specifically assess formal coordination mechanisms or decision‐making processes within institutions. Future research could improve understanding of community perceptions by using institutional network analysis or policy implementation studies to obtain a more comprehensive understanding of collaboration dynamics.

## Funding

This research did not receive any specific grant from funding agencies in the public, commercial, or not‐for‐profit sectors.

## Conflicts of Interest

The authors declare no conflicts of interest.

## Supporting Information

Additional supporting information can be found online in the Supporting Information section.

## Supporting information


**Supporting Information** Appendix 1: This file provides definitions of all abbreviations and acronyms used throughout the manuscript to aid reader comprehension.

## Data Availability

The data that support the findings of this study are available from the corresponding author upon reasonable request.
