# Supplementary material for: Local Community Perspectives on the Role of Institutional Stakeholders in Advancing Forest Landscape Restoration in Ghana
Source: Scientifica (Cairo). 2026 May 30;2026:3179536. doi: 10.1155/sci5/3179536 (PMC13239224; doi:10.1155/sci5/3179536)
Supplement: Supplementary file 1 — Supporting Information Appendix 1: This file provides definitions of all abbreviations and acronyms used throughout the manuscript to aid reader comprehension. [file SCI5-2026-3179536-s001.docx]

Supplementary Material

Appendix 1. List of Abbreviations

| Abbreviation | Definitions |
| --- | --- |
| FLR | Forest Landscape Restoration |
| EPT | Environmental Perception Theory |
| LBC | Licensed Buying Companies |
| COCOBOD | Cocoa Board, Ghana |
| FGD | Focus Group Discussions |
| NGOs | Non-governmental Organizations |
| FC | Forestry Commission |
| FLEGT | Forest Law Enforcement, Governance, and Trade |
| REDD+ | Reducing Emissions from Deforestation and Forest Degradation |
